# Relationship between meibomian gland loss in infrared meibography and meibum quality in dry eye patients

**DOI:** 10.1186/s12886-022-02509-5

**Published:** 2022-07-04

**Authors:** Minji Ha, Si-Eun Oh, Woong-Joo Whang, Kyung-Sun Na, Eun Chul Kim, Hyun-Seung Kim, Jin Soo Kim, Ho Sik Hwang

**Affiliations:** 1 Department of Ophthalmology, College of Medicine, The Catholic University of Korea, 222, Banpo-daero, Seocho-gu, Seoul, Republic of Korea; 2 Department of Ophthalmology, Yeouido St. Mary’s Hospital, The Catholic University of Korea, 10, 63-ro, Yeongdeungpo-gu, Seoul, 07345 Republic of Korea; 3 Department of Ophthalmology, Chuncheon Sacred Heart Hospital, Hallym University, 77, Sakju-ro, Chuncheon-si, Gangwon-do Republic of Korea

**Keywords:** Meibum, Meibum quality, Meibomian gland dysfunction, Dry eye disease, Meiboscore

## Abstract

**Background:**

In the present study, we evaluated the correlation between meibomian gland dropout and meibum quality in the same central 8 meibomian glands of the eyelid.

**Methods:**

Ninety-nine eyes of 91 patients with dry eye were included in the study. Dropout of the 8 central meibomian glands of the eyelids was graded as 0, 1, 2, or 3, according to the dropout area. The meibum quality was graded as follows: grade 0, no secretion; 1, inspissated/toothpaste consistency; 2, cloudy liquid secretion; and 3, clear liquid secretion. For 68 eyes of 68 patients, correlation analysis between dropout and meibum quality was performed. To precisely analyze the direct correlation between meibomian gland dropout in meibography and meibum quality, we evaluated 31 eyes of 23 patients with focal dropout in meibography.

**Results:**

The median (interquartile range) meiboscore was 1.0 (2.0) in the upper eyelids and 0.0 (1.0) in the lower eyelids. The median (interquartile range) meibum quality grade was 3.0 (1.0) in the upper eyelids and 1.0 (1.0) in the lower eyelids. No significant correlation between the meiboscore and meibum quality grade was detected in the upper (*p* =0.746) or lower (*p* =0.551) eyelids. Analysis of the direct correlation between meibomian gland dropout in meibography and meibum quality in patients with focal dropout (loss of 1 or 2 adjacent meibomian glands), however, indicated that meibomian glands with dropout secreted little to no meibum.

**Conclusions:**

Overall analysis revealed no relationship between meibomian gland dropout and meibum quality, but more detailed investigation of each meibomian gland alone revealed that meibomian glands with dropout secrete little to no meibum.

**Supplementary Information:**

The online version contains supplementary material available at 10.1186/s12886-022-02509-5.

## Background

Dry eye is a multifactorial disease of the ocular surface characterized by a loss of homeostasis of the tear film, and accompanied by ocular symptoms, in which tear film instability and hyperosmolarity, ocular surface inflammation and damage, and neurosensory abnormalities play etiological roles [1]. An evaporative component to dry eye disease is more common than an aqueous deficient component [2, 3]. Meibomian gland dysfunction (MGD), a contributor to evaporative dry eye, is considered the leading cause of dry eye in clinic and population based studies [3–5].

Meibomian gland dysfunction (MGD) is defined as a chronic, diffuse abnormality of the meibomian glands, commonly characterized by terminal duct obstruction and/or qualitative/quantitative changes in glandular secretion [6]. MGD is a major cause of evaporative dry eye disease [7].

To diagnose MGD clinically, ophthalmologists observe the eyelid margin, including the orifices of the meibomian glands, and evaluate the amount and quality of the meibum secreted from the meibomian gland orifices by squeezing the eyelids [8]. In the healthy eyelid, meibum is clear and easily expressed with gentle digital pressure [9]. However, the quality of meibum in patients with MGD is varied. In MGD patients, meibum can lose its clarity to become cloudy and then opaque and its viscosity can be increased, becoming toothpaste-like and hard to express in eyelids with severe MGD [9]. The qualities of meibum and its expressibility have been evaluated in various grading schemes [10]. Since the introduction of noncontact infrared meibography by Reiko Arita [11], noncontact infrared meibography has been widely used in clinics to directly evaluate meibomian gland morphology [10, 12, 13]. Normal meibomian gland morphology and the absence of meibomian gland loss (dropout) in meibography might lead clinicians to assume that a normal amount of clear meibum will be secreted from the meibomian glands by eyelid squeezing. Similarly, if extensive dropout is observed, clinicians might assume that only a scant amount of meibum with increased viscosity will be secreted from the meibomian glands by eyelid squeezing. In actual clinical practice, however, we often encounter patients whose characteristics are not consistent with these predictions, and therefore, some ophthalmologists may to conclude that meibomian gland dropout in meibography does not correlate with the meibum quality.

In the present study, we analyzed the correlation between meibography and meibum quality. In addition, meibum quality and expression were investigated in glands with focal dropout. This allowed us to evaluate the direct relationship between meibography and meibum quality in these cases.

## Methods

### Participants

This study included 99 eyes of 91 patients who visited Chuncheon Sacred Heart Hospital for dry eye examination. The inclusion criteria were age of ≥20 years and at least mild dry eye symptoms (an Ocular Surface Disease Index [OSDI] score ≥13) and low tear film break-up time (TBUT <5 s) using fluorescein dye, or a low Schirmer I score (<10 mm /5 min without anesthesia), or corneal punctate fluorescein staining (Oxford staining score >1) [14] in at least 1 eye. The exclusion criteria were (1) history of ocular injury; (2) eyelid infection; (3) ocular surgery within the previous 6 months; (4) non–dry-eye ocular inflammation; (5) uncontrolled systemic disease; and (6) patients who were unable to undergo infrared meibography or lid squeezing.

### Relationship between meibomian gland loss in infrared meibography and meibum quality in the same central 8 meibomian glands of the eyelid

**Fig. 1:**
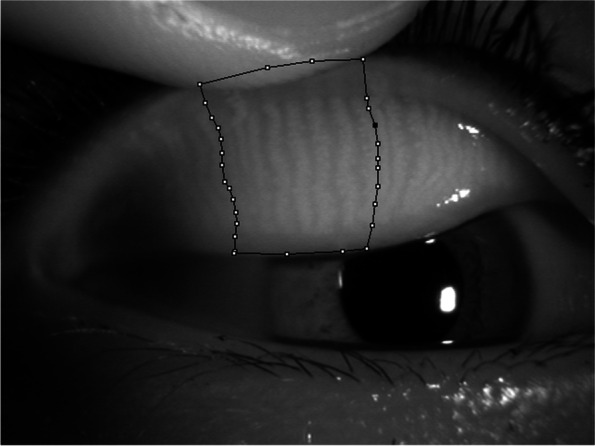
Meibomian gland dropout scoring. After infrared meibography, meibomian gland dropout in the central 8 meibomian glands of the eyelid was scored (meiboscore), as follows. For the central 8 meibomian glands of the eyelid: score 0, no dropout; score 1, less than one-third dropout; score 2, more than one-third dropout, but less than two-thirds dropout; and score 3, more than two-thirds dropout

For 68 patients, meibography photographs were obtained using a slit-lamp microscope (Slit Lamp BQ 900; Haag-Streit, Köniz, Switzerland; 10x magnification) equipped with an infrared filter (R-72, cut-on wavelength 720 nm, Edmund optics, Barrington, NJ) and an infrared camera (acA1600-20um; Basler Inc., Ahrensburg, Germany) [15]. After infrared meibography, meibomian gland dropout in the central 8 meibomian glands of the eyelid was scored (meiboscore; Fig. 1). The area of gland dropout was defined according to Pult et al. by “(1) the actual ending of glands, (2) the width of the area, defined to be between at least from the tear punctum, and the temporal border defined to be to the most well visible tarsal conjunctiva of the everted lid, and (3) the maximal depth of the area was estimated to be where glands would have ended in normal meibomian gland morphology [16–18]. For the central 8 meibomian glands of the eyelid: score 0, no dropout; score 1, less than one-third dropout; score 2, more than one-third dropout, but less than two-thirds dropout; and score 3, more than two-thirds dropout. These criteria are a modification of those described by Arita et al. [11]. Scoring of the meibomian gland dropout was performed by 1 investigator (HHS) with experience and expertise in grading meibomian glands.

After performing infrared meibography, the meibum quality was evaluated. A drop of topical anesthesia was instilled into the inferior fornix. The upper and lower eyelids were squeezed between a cotton swab and the examiner’s finger. Squeezing of the eyelids was performed by 1 investigator (HHS) and the pressure applied was kept as similar as possible. The squeezing and meibum secretion were recorded using a color charge-coupled device camera (Guppy, Allied Vision, Exton, PA) connected to a slit-lamp.

After completing and saving all of the recordings, meibum secretion from 68 upper eyelids and 68 lower eyelids was evaluated in 1 day. The meibum quality was graded as follows: grade 0, no secretion; 1, inspissated/toothpaste consistency, 2: cloudy liquid secretion, and 3: clear liquid secretion [19]. Grading of the meibum quality was performed by the same investigator (HHS). Prior to grading, however, the investigator scored the meibomian gland dropout in meibography of 68 patients in a single day without watching the videos showing the eyelid squeezing. After 1 week, the investigator graded the meibum quality by watching the videos of 68 patients showing the eyelid squeezing without reviewing the meibography, and recorded the meibum quality grade in a separate spreadsheet for masking.

To analyze the direct correlation between meibomian gland dropout and meibum quality as accurately as possible, meibomian gland dropout and meibum quality were evaluated in the same central 8 meibomian glands of the eyelid. In case of varying meibum quality within the central 8 meibomian glands of the eyelid, the score of the dominant meibum quality was used as the meibum quality grade. Only right eye data were used in this analysis.

### Meibum secretion from orifices corresponding to focal meibomian gland dropout

**Fig. 2:**
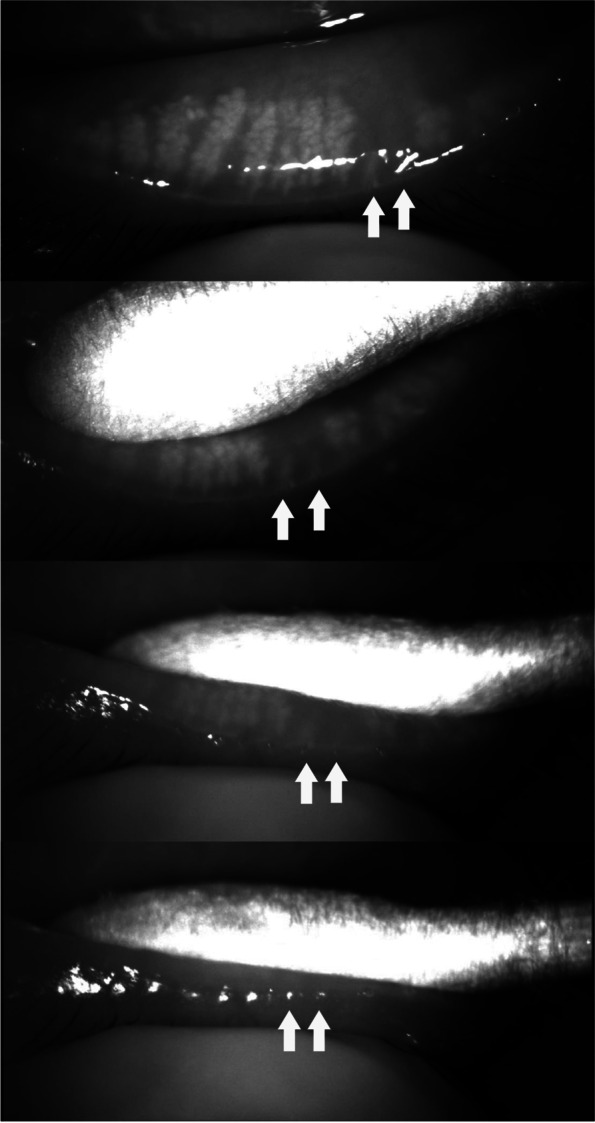
Meibum secretion from the orifices of two adjacent meibomian glands in the area of focal dropout (two arrows). To precisely analyze the direct correlation between meibomian gland dropout in meibography and meibum quality, we evaluated 31 eyes of 23 patients with focal dropout (loss of 1 or 2 adjacent meibomian glands) in meibography. While squeezing the eyelid in the region of the focal dropout, we recorded the meibomian glands and the secretion with an infrared charge-coupled device infrared video camera (acA1600-20um; Basler Inc., Ahrensburg, Germany). The color of the meibum was visually evaluated through a slit-lamp microscope. Using this procedure, we checked the meibum secretion from the orifices of 1 or 2 adjacent meibomian glands in the region of the focal dropout

To precisely analyze the direct correlation between meibomian gland dropout in meibography and meibum quality, we evaluated 31 eyes of 23 patients with focal dropout (loss of 1 or 2 adjacent meibomian glands) in meibography. While squeezing the eyelid in the region of the focal dropout, we recorded the meibomian glands and the secretion with an infrared charge-coupled device infrared video camera (acA1600-20um; Basler Inc., Ahrensburg, Germany) (Fig. 2, Video 1). The color of the meibum was visually evaluated through a slit-lamp microscope. Using this procedure, we checked the meibum secretion from the orifices of 1 or 2 adjacent meibomian glands in the region of the focal dropout. In these cases, we assessed not only the meibum quality, but also the meibum quantity according to the following criteria [20]: Upon firm digital pressure, volume of expressed meibum, 0: normal volume – just covers the orifice; 1: volume 2- to 3-fold greater than normal; 2: volume 4- to 9-fold greater than normal; 3: volume 10-fold greater than normal.

### Statistical Analysis

We performed a normality test for meiboscores and meibum quality grades in the upper and lower eyelids using the Kolmogorov-Smirnov test. Because none of the grades showed a normal distribution (*p*=0.000, all), we used non-parametric tests for statistical analysis of the meiboscores and meibum quality grades.

The median (interquartile range) meiboscores of the upper and lower eyelids were obtained and a Wilcoxon signed rank test was performed to compare the mean meiboscore between the upper and lower eyelids. The median (interquartile range) meibum quality grades of the upper and lower eyelids were obtained, and a Wilcoxon signed rank test was performed to compare the mean meibum quality grade between the upper and lower eyelids. Correlation analysis between meibomian gland dropout (meiboscore) and the meibum quality grade was performed for the 68 upper and 68 lower eyelids with the Spearman’s rank correlation test and the Chi-squared test. The results were considered statistically significant when *p* < 0.05. For statistical analysis, IBM SPSS 24.0 software (IBM Corp., Armonk, NY) was used.

## Results

### Relationship between meibomian gland loss in infrared meibography and meibum quality in the central 8 meibomian glands of the eyelids

Table 1 shows the demographic data of the 68 patients included in the correlation analysis of the meiboscore and meibum quality in the central 8 meibomian glands of the eyelids. In the central 8 meibomian glands of the eyelid, the median meiboscore was 1.0 in the upper eyelids and 0.0 in the lower eyelids. The dropout was significantly greater in the upper eyelids than in the lower eyelids (*p*=0.010). The median meibum quality grade was 3.0 in the upper eyelids and 1.0 in the lower eyelids. The meibum quality grade was significantly lower in the lower lid than in the upper eyelid (*p*=0.000).

**Table 1:** Demographic characteristics of the patients included in the study, meiboscore and meibum quality score in upper and lower eyelids

Characteristics	Value (median (interquartile range))		
Age (years)	58.0 (13.5)		
Sex	Male: 18 (27%) Female: 50 (73%)		
Number of patients	68		
Number of eyes	68		
TBUT (s)	9.6 (12.6)		
Schirmer test (mm)	7.0 (9.5)		
Corneal stain (Oxford scale)	0.0 (2.0)		
OSDI	38.64 (43.44)		
	Upper eyelids	Lower eyelids	*p*-value
Meiboscore^†^	1.0 (2.0)	0.0 (1.0)	0.010^*^
Meibum quality score^‡^	3.0 (1.0)	1.0 (1.0)	0.000^*^

*TBUT* Tear break-up time, *OSDI* Ocular surface disease index

^†^Meiboscore: For the central eight meibomian glands in the eyelid: score 0, no dropout; score 1, dropout less than 1/3; score 2, more than 1/3 and less than 2/3 dropout; and score 3, more than 2/3 dropout

^‡^Meibum quality score: The meibum quality was graded as follows: 0: no secretion; 1: inspissated/toothpaste consistency; 2: cloudy liquid secretion; and 3: clear liquid secretion

^*^Wilcoxon signed rank test

Table 2 shows the correlation between meibomian gland dropout and meibum quality in the upper eyelids. In the 29 eyelids with no dropout, the meibum quality was ‘no meibum’ in 1, ‘inspissated/toothpaste consistency’ in 3, ‘cloudy liquid secretion’ in 8, and ‘clear liquid secretion’ in 17. In the 11 eyelids having a meiboscore of 3 with greater than two-thirds dropout, the meibum quality was ‘no meibum’ in 1, ‘inspissated/toothpaste consistency’ in 3, ‘cloudy liquid secretion’ in 1, and ‘clear liquid secretion’ in 6. Statistically, there was no significant correlation between the meiboscore and the meibum quality grade in the upper eyelids (Spearman’s rank correlation test, *p*=0.746, Chi-squared *p*=0.451).

**Table 2:** The distribution of the meibum quality from the central eight meibomian glands in the upper eyelids according to the dropout (meiboscore) at central eight meibomian glands in the upper eyelids

Upper eyelid		Meibum quality from the central eight meibomian glands in the eyelids				
		No meibum	Inspissated/toothpaste consistency	Cloudy liquid secretion	Clear liquid secretion	Total
Meiboscore at the central eight meibomian glands in the eyelids	0	1	3	8	17	29
	1	0	5	4	6	15
	2	0	1	3	9	13
	3	1	3	1	6	11
	Total	2	12	16	38	68

Spearman’s rank correlation test, *p*=0.746

Chi-squared test, *p*=0.451

Table 3 shows the correlation between meibomian gland dropout and meibum quality in the lower eyelids. Overall, ‘clear liquid secretion’ meibum quality was observed less frequently and ‘inspissated/toothpaste consistency’ were observed more frequently (‘clear liquid secretion’, 4 eyes; ‘inspissated/toothpaste consistency’, 47 eyes) compared with the upper eyelid. Among the 42 eyelids with a meiboscore of 0, ‘inspissated/toothpaste consistency’ was observed in 30, ‘cloudy liquid secretion’ was observed in 9, and ‘clear liquid secretion’ was observed in 3. In the 5 eyelids with a meiboscore of 3, ‘inspissated/toothpaste consistency’ was observed in 4 and ‘clear liquid secretion’ was observed in 1. There was no statistically significant correlation between the meiboscore and meibum quality grade in the lower eyelids (Spearman’s rank correlation test, *p*=0.551, Chi-squared *p*=0.379).

**Table 3:** The distribution of the meibum quality from the central eight meibomian glands in the lower eyelids according to the dropout (meiboscore) at central eight meibomian glands in the lower eyelids

Lower eyelid		Meibum quality from the central eight meibomian glands in the eyelids				
		No meibum	Inspissated/toothpaste consistency	Cloudy liquid secretion	Clear liquid secretion	Total
Meiboscore at the central eight meibomian glands in the eyelids	0	0	30	9	3	42
	1	1	7	3	0	11
	2	1	6	3	0	10
	3	0	4	0	1	5
	Total	2	47	15	4	68

Spearman’s rank correlation test, *p*=0.551

Chi square test, *p*=0.379

### Meibum secretion from orifices corresponding to focal meibomian gland dropout

**Fig. 3:**
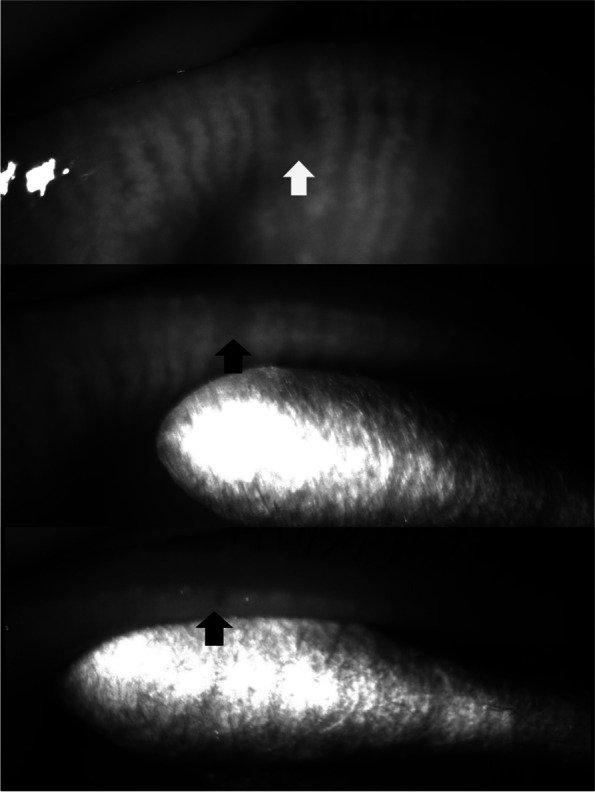
A case (F/55) in which meibum was not secreted from the orifice of a meibomian gland in the area of focal dropout (arrow)

**Fig. 4:**
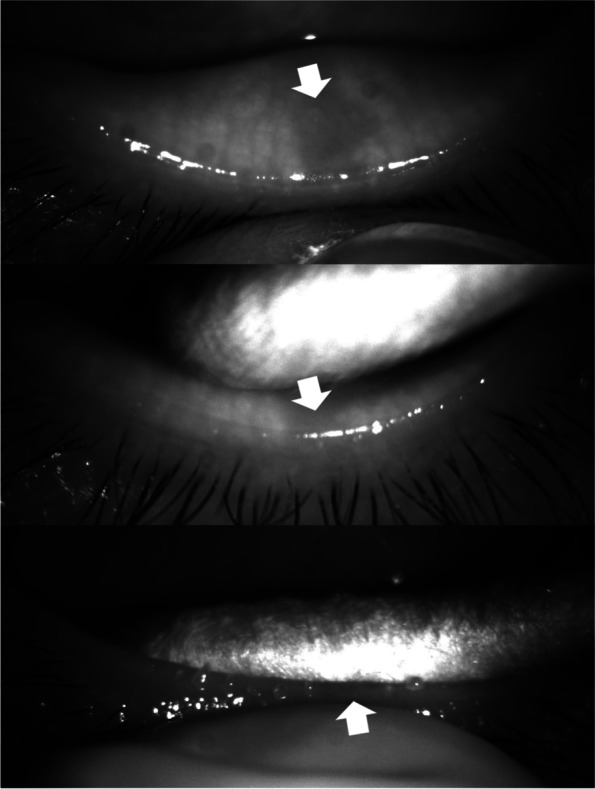
A case (F/51) in which meibum was not secreted from the orifice of a meibomian gland in the area of focal dropout (arrow)

**Fig. 5:**
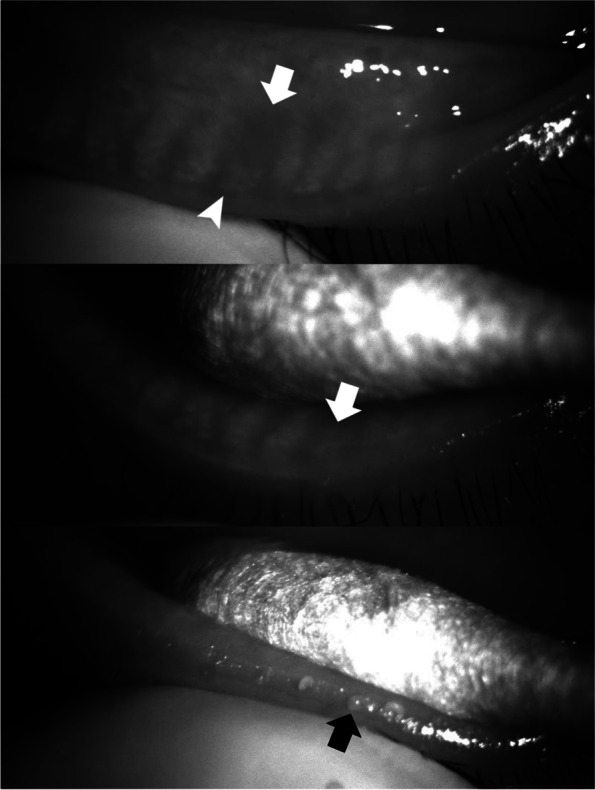
A case (M/42) in which a normal volume of meibum (just covers orifices) with cloudy liquid was secreted at the site with focal meibomian gland loss (arrow). In this case, the dropout of the meibomian gland was not complete, and some acini (arrow head) remained near the orifice

**Fig. 6:**
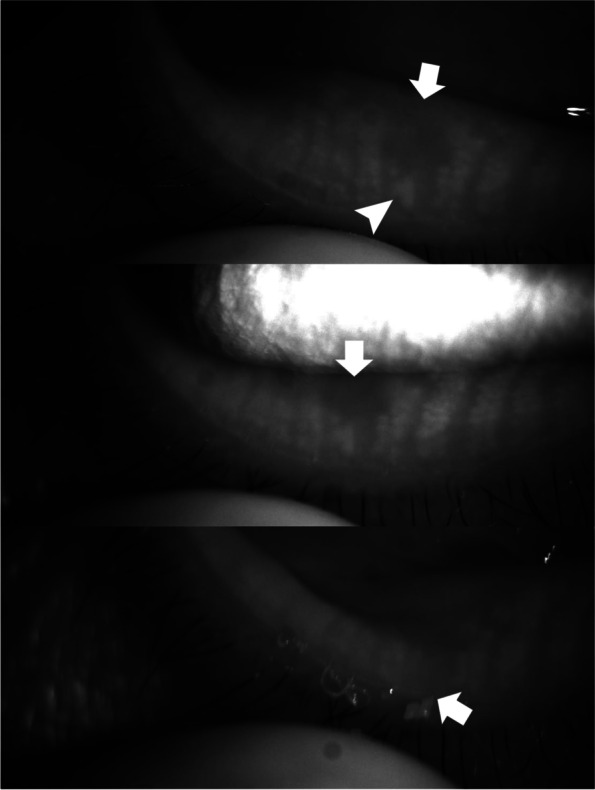
A case (F/49) in which an increased volume of meibum 2 to 3 times greater than normal with an inspissated consistency was secreted at the site with focal meibomian gland loss (arrow). In this case, the dropout of the meibomian gland was not complete, and some acini (arrow head) remained near the orifice

Focal dropout (1 or 2 meibomian glands) in meibography was observed in 31 eyelids of 23 patients. The median (interquartile range) age was 56.0 (9.0) years. Among the 31 eyelids, 2 (6%) were upper eyelids and 29 (94%) were lower eyelids. Among the 31 eyelids, 26 eyelids (84%) showed no meibum secretion from the orifice(s) (Figs. 3, 4, Videos 2, 3). In the remaining 5 eyelids (16%), 4 eyes showed a normal volume (just covers orifices) with ‘cloudy liquid’ and 1 eye showed an increased volume 2- to 3-fold greater than normal with an inspissated consistency (Figs. 5, 6, Videos 4, 5). In these cases, the dropout of the meibomian glands was not complete, and some meibomian gland acini tissue remained near the orifices.

## Discussion

In this study, to determine the correlation between meibomian gland loss (dropout) and meibum quality, we evaluated as accurately as possible the correlation between meibomian gland dropout and meibum quality in the same central 8 meibomian glands of the eyelid. Furthermore, we analyzed the direct correlation between meibomian gland dropout and meibum quality by analyzing 31 eyes of 23 patients with focal dropout (loss of 1 or 2 adjacent meibomian glands) in meibography. When squeezing the eyelid in the region of the focal dropout, we recorded the meibomian glands and the meibum secretion with an infrared camera. This procedure allowed us to grade the meibum secretion from the orifices of 1 or 2 adjacent meibomian glands.

Analysis of the central 8 meibomian glands of the eyelids in 68 eyes revealed no significant correlation between meibomian gland dropout and meibum quality in the upper and lower eyelids (Tables 2 and 3), which is often observed in the clinic. On the basis of this finding alone, therefore, clinicians may underestimate the clinical value of infrared meibography. The lack of a significant correlation between these 2 measurements, however, may be explained as follows. First, we evaluated only the central 8 meibomian glands of the eyelid to assess the correlation as accurately as possible. Even within this specific region, dropout appeared differently depending on the location, and the quality of the meibum secreted by each orifice also varied depending on the location. For example, in an eyelid with less than one-third dropout (meiboscore 1) at the temporal side, but clear meibum secreted from the middle and nasal side, the overall meibum quality would be graded ‘clear liquid secretion’ even if the meiboscore is 1. Second, the meibum quality will be normal if there is no MGD. If the MGD is hypersecretory, however, meibum secretion will be increased and the meibum may be clear or yellow with or without increased viscosity [6]. If there is obstructive MGD, either meibum with toothpaste consistency or no meibum at all will be secreted [6]. As such, when there is no dropout, the meibum quality will vary depending on the state of the meibomian glands. Third, even if there is dropout, if a part of the meibomian gland remains near the orifices (shortening), the meibum quality will vary.

Analysis of the direct correlation between meibomian gland dropout and meibum quality in 31 eyes of 23 patients with focal dropout (i.e., loss of 1 or 2 adjacent meibomian glands) in meibography revealed that, among the 31 eyelids, 26 (84%) did not secrete meibum from the orifice(s), 4 eyes showed a normal volume (just covers orifices) with cloudy liquid, and 1 eye showed a volume 2- to 3-fold greater than normal with an inspissated consistency. That is, in meibomian glands with dropout, there was little to no secretion of meibum from the meibomian gland.

Eom et al. reported that meibomian gland dropout observed in meibography correlated with the quality of the meibum secreted after eyelid expression in 26 eyes with obstructive MGD [21]. The meibum quality was observed in 8 meibomian glands located in the middle third of the upper and lower eyelids, whereas infrared meibography was used to measure the meibomian gland dropout area for the middle two-thirds, and the ratio was calculated. They reported that the greater the loss of the meibomian glands in both the upper and lower eyelids, the higher the value of the meibum quality grade (i.e., increased viscosity or toothpaste consistency). One difference between our study and that of Eom et al. is that we selected the same central 8 meibomian glands for both measurements, and not the middle two-thirds region for infrared meibography and the central 8 meibomian glands for meibum quality. Because dropout is not uniform across the entire eyelid and the quality of the meibum secreted by squeezing is not uniform across the entire eyelid, it is preferable to analyze a same small area. We did our best to accurately analyze the direct correlation between dropout and meibum quality by choosing the same central 8 meibomian glands. Second, we analyzed the direct correlation between meibomian gland dropout and meibum quality in 23 patients with focal dropout in meibography. Third, unlike Eom et al., our results did not demonstrate a significant correlation between dropout and meibum quality in 68 eyes of 68 patients. Our findings are consistent with our observations in the clinic. Fourth, Eom et al. analyzed 26 eyes of patients with obstructive MGD, and we analyzed 99 eyes of 91 patients who visited our clinic for dry eye disease.

We found that meibomian glands are more often obstructed in the lower eyelids than in the upper eyelids. Table 1 shows that the upper eyelids had a higher meiboscore, but the lower eyelids had a lower meibum quality grade (inspissated/toothpaste consistency or no meibum). Therefore, the obstructive type of MGD is considered to be more prevalent in the lower eyelid. Eom et al. reported similar results in their study, in which the quality of expressed meibum was assessed on a scale of 0 to 3 for each gland: 0 = clear; 1 = cloudy; 2 =cloudy with debris; 3 = inspissated (toothpaste-like) [21]. Complete obstruction without meibum expression when applying firm digital pressure was graded as a 3. Meibum quality was assessed in 8 glands in the central third area of the upper and lower eyelids (total score range, 0–24). The mean expressed meibum grade (±SD) of the lower eyelids was 16.5 ± 5.1, which was significantly greater than that of the upper eyelids (11.2 ± 5.2) [21]. In another study, Eom et al. reported that the mean number of glands expressible among the central 8 glands in the upper eyelids was 3.9 ± 2.6, significantly higher than that for the lower eyelids (2.2 ± 2.4, *p* < 0.001) [22]. The upper eyelids move more than the lower eyelids during blinking. Therefore, meibum in the upper eyelids appears to be more easily and continuously secreted by the mechanical action of the pretarsal orbicularis muscle, making it less clogged than the lower eyelids [23, 24].

In this study, the dominant meibum quality score was used as the meibum quality grade in cases of varying meibum quality within the central 8 meibomian glands of the eyelid. Some studies report the highest grade found among the expressed glands [16, 25]. It would be ideal to use the average meibum quality score from 8 meibomian glands, however, instead of the meibum quality score of the dominant meibomian gland.

We performed the eyelid squeezing last because it is an invasive procedure. Eyelid manipulation during meibography may inadvertently lead to the expression of meibum and confound the meibum quality assessment. Because we applied firm pressure for eyelid squeezing instead of gentle pressure, however, we do not believe that eyelid manipulation during meibography prior to eyelid squeezing confounded the observer’s ability to assess the meibum quality.

This study have some limitations. First, we did not calculate the sample size before the study. Second, only patients with dry eye were included in the study. Third, we analyzed only the central Meibomian glands in the eyelid.

## Conclusions

The present study revealed no significant correlation between meibomian gland dropout in meibography and meibum quality in the central 8 meibomian glands of the upper and lower eyelids. Analysis of the direct correlation between meibomian gland dropout in meibography and meibum quality in patients with focal dropout (loss of 1 or 2 adjacent meibomian glands), however, indicated that meibomian glands with dropout secrete little to no meibum. In other words, macroscopically, there appears to be no relationship between meibomian gland dropout and meibum quality, but more detailed observation of each meibomian gland alone reveals that meibomian glands with dropout secrete little to no meibum.

## Supplementary Information

**Additional file 1:** **Video 1**. Meibum secretion from the orifices of two adjacent meibomian glands in the area of focal dropout (two arrows). To precisely analyze the direct correlation between meibomian gland dropout in meibography and meibum quality, we evaluated 31 eyes of 23 patients with focal dropout (loss of 1 or 2 adjacent meibomian glands) in meibography. While squeezing the eyelid in the region of the focal dropout, we recorded the meibomian glands and the secretion with an infrared charge-coupled device infrared video camera (acA1600-20um; Basler Inc., Ahrensburg, Germany). The color of the meibum was visually evaluated through a slit-lamp microscope. Using this procedure, we checked the meibum secretion from the orifices of 1 or 2 adjacent meibomian glands in the region of the focal dropout.

**Additional file 2:** **Video 2**. A case (F/55) in which meibum was not secreted from the orifice of a meibomian gland in the area of focal dropout.

**Additional file 3:** **Video 3**. A case (F/51) in which meibum was not secreted from the orifice of a meibomian gland in the area of focal dropout.

**Additional file 4:** **Video 4**. A case (M/42) in which a normal volume of meibum (just covers orifices) with cloudy liquid was secreted at the site with focal meibomian gland loss (arrow). In this case, the dropout of the meibomian gland was not complete, and some acini (arrow head) remained near the orifice.

**Additional file 5:** **Video 5**. A case (F/49) in which an increased volume of meibum 2 to 3 times greater than normal with an inspissated consistency was secreted at the site with focal meibomian gland loss (arrow). In this case, the dropout of the meibomian gland was not complete, and some acini (arrow head) remained near the orifice.

## Data Availability

The datasets used and/or analyzed during the current study are available from the corresponding author on reasonable request.

## Abbreviations

MGD: Meibomian gland dysfunction
IRB: Institutional Review Board
OSDI: Ocular Surface Disease Index
TBUT: Tear film break-up time
SD: Standard deviation
